# Examine the relationships between health-related quality of life, achievement motivation and job performance: the case of Taiwan hospitality industry

**DOI:** 10.1186/s40359-022-00884-8

**Published:** 2022-07-13

**Authors:** Wei-Ya Ni, Eric Ng, Yi-Te Chiang, Ben A. LePage, Feng-Hua Yang, Wei-Ta Fang

**Affiliations:** 1 Ph.D. Program in Management, Da-Yeh University, No.168, University Rd., Dacun, Changhua, 51591 Taiwan, ROC; 2 School of Business, University of Southern Queensland, Toowoomba, QLD 4350 Australia; 3 Graduate Institute of Environmental Education, National Taiwan Normal University, Taipei, 11677 Taiwan, ROC; 4 Academy of Natural Sciences, 1900 Benjamin Franklin Parkway, Philadelphia, PA 19103 USA; 5 Department of International Business Management, Da-Yeh University, No.168, University Rd., Dacun, Changhua, 51591 Taiwan, ROC

**Keywords:** Achievement motivation, COVID-19, Hospitality, HRQoL, Job performance, Quality of life

## Abstract

**Background:**

Employees are considered as one of the most important assets in many organizations, and their health well-being is critical to help achieve a sustainable and motivated workforce that is committed to delivering quality hospitality services through enhanced performance and productivity. Given the extent of the challenges and impact presented by the COVID-19 pandemic to the hospitality industry, it is timely to gain further insights on employees’ health well-being. The key purpose of this study is to examine the relationships between health-related quality of life, achievement motivation and job performance in the Taiwan hospitality industry, to acquire a better understanding of their relationships through the job performance pathway models.

**Methods:**

This study has used a purposeful sampling technique to select the 10 highest-earning hospitality companies in Taiwan. A total of 292 questionnaires were collected from the employees of these hospitality companies. Based on the multi-dimensional concept of health-related quality of life (HRQoL), the relationships between the five key dimensions (i.e. psychological health, physical health, social health, achievement motivation, and job performance) were examined. To measure these dimensions, the survey questions were adapted from previous research such as the World Health Organization’s WHOQOL-BREF scale, Minnesota Satisfaction Questionnaire. Partial least squares - Structural Equation Modeling method was used to explore these dimensions, and two job performance pathway models (for manager and staff) were subsequently developed.

**Results and conclusions:**

Findings showed that psychological health directly affected the manager’s job performance and physical health had a similar effect through social health. While psychological health had not affected the staff’s job performance, but it could affect achievement motivation through both direct and indirect effects of social health. The pathway models that were developed indicated that the manager’s job performance was mainly affected by psychological health and social health, whereas the key dimension that had affected the staff’s job performance was achievement motivation.

## Background

The outbreak of COVID-19 pandemic has brought unprecedent issues and challenges that have significantly impacted the economy, society, and lifestyle globally. One of the worst affected is the hospitality industry, which underpins many economic activities that plays an important role contributing towards economic and employment growth. According to the World Travel and Tourism Council, the hospitality industry is considered a key driver in global value creation and has contributed about USD$8.8 trillion to the global economy in 2018 [1]. During the COVID-19 period, this labor-intensive industry has seen constant evolving changes, significant decreases in jobs as well as changes in their roles, which have an enormous impact on the workforce.

Employees are often regarded as one of the most important assets in many organizations, and this is particularly evident in the hospitality industry that requires high level of interpersonal interactions where service quality and delivery as well as customer satisfaction are critical indicators to the business success [2]. Thus, employees’ health well-being is very important to help attain a sustainable and motivated workforce.

Health-related quality of life (HRQoL) is a multi-dimensional concept that involves several domains associated with physical, psychological, and social functioning that focuses on the impact of health well-being of a person’s ability to live a fulfilling life [3, 4]. In the past, HRQoL has been used in different fields and contexts to investigate academic performance [5], work type [6], health systems evaluation [7], work related stress [8], age group [9]. But despite the high level of interpersonal interactions characterized in the hospitality industry, limited studies have been conducted to explore the relationships between HRQoL, achievement motivation and job performance that can affect the delivery of quality hospitality services.

Ford et al. [10] define job performance as a person’s behavior and skills, and the extent to which these help a business achieve its goals. Highly skilled employees are more likely to achieve better job performance and therefore play a critical role in contributing to the organization’s success. Over the years, hospitality organizations have been constantly reviewing their management techniques and principles to improve the quality of life of their employees so that organizations can be better positioned to achieve their desired goals [11]. There is evidence that physical, psychological, and social health are important for enhancing job performance [10, 12].

Another organizational behavior factor that is associated with job performance is achievement motivation [13–15]. Achievement motivation is the need for excellence to attain significant accomplishment on the tasks performed and achieving aspiration in life [16, 17]. In the hospitality industry, employees’ achievement motivation is relatively low since the sector is characterized by low wages, irregular and long working hours, and high staff turnover [18, 19]. There is evidence indicating that employees who exhibit a high level of achievement motivations are more likely to engage in their work and achieve higher job performance through constant challenges that are attainable [16, 20]. Interpersonal interactions between managers and employees are often an ongoing and complex issue in many organizations, especially when it involves managers having to create, encourage and increase staff motivation to enhance their job performance [21]. It is also noted that differences between management and staff inevitably have an impact on many aspects of an organization, which may include communication, performance of tasks, and the functioning of the organization. Research has found conceptual differences that contribute to gaps between managers and staff [22], and thus further research is needed to gain a better understanding of the differing perspectives.

Psychological health as an individual’s emotional development experience [4] can result in cognitive problems that affect job performance [23, 24] in a personal as well as an organizational context [25–27]. According to Ford et al. [10], there is a moderate to strong correlation between psychological health and job performance. For example, poor psychological health and stress can negatively affect employees’ job performance, productivity, and overall morale, which will have an adverse impact on the business profitability and competitiveness in the hospitality industry. Therefore, the following hypotheses are proposed:

### Hypothesis 1a

Psychological health has a significant positive effect on job performance for managers.

### Hypothesis 1b

Psychological health has a significant positive effect on job performance for staff.

Social health considers an individuals’ interpersonal relationships and role performance [4] and entails the fairness of interpersonal treatment [28]. Previous studies suggest that psychological health is associated with social interaction [29], interpersonal relationship disturbances [30], and interpersonal relationship problems [31]. Furthermore, psychological disorders such as depression may also cause interpersonal relationship disturbances [32]. Thus, there is evidence that indicates a possible correlation between psychological and social health, especially in the hospitality industry that is characterized by interpersonal interactions. As such, the following hypotheses are proposed:

### Hypothesis 2a

Psychological health has a significant positive effect on the social health of managers.

### Hypothesis 2b

Psychological health has a significant positive effect on the social health of staff.

Studies have shown that psychological health is associated with achievement motivation [33–35]. For example, research conducted by Deary et al. [36] about consultant doctors working in Scotland reveal that personal achievement is related to job stress, which can further affect job burnout. Other cross-sectional studies have also investigated the relationship between psychological health and achievement motivation for high school students in the mental health aspects of depression and self-esteem [37], assess relationships between mental health, achievement motivation, and academic success for Kurdistan university students [38], determine the effect of emotional intelligence on achievement motivation, and psychological changes of secondary school students in India [39], explore aspects of health and dietary habits on achievement motivation of college students with ADHD in America [40]. Although the causal relationship between psychological health and achievement motivation has not been conclusively established, the association between the two is clear. Therefore, the following hypotheses are proposed:

### Hypothesis 3a

Psychological health has a significant positive effect on achievement motivation for managers.

### Hypothesis 3b

Psychological health has a significant positive effect on achievement motivation for staff.

Testa and Simonson [4] state that physical health includes a person’s physical functioning as well as symptoms of disability or disease. There have been numerous studies conducted on physical health to investigate its relationship with different fields of studies which include emotional states [41], social capital and support [42, 43], alcohol consumption [44], domestic violence [45]. More importantly, there is also evidence to suggest that physical health is correlated with work and job performance [10, 46]. Given the importance of HRQoL on job performance [12], the following hypotheses are proposed:

### Hypothesis 4a

Physical health has a significant positive effect on job performance for managers.

### Hypothesis 4b

Physical health has a significant positive effect on job performance for staff.

Research has found that poor physical functioning produces higher social stress and lower social integration [47]. Furthermore, studies reveal that deficiencies in social skills are closely associated with physical health [48], whereas physical illness can also have a negative impact on interpersonal relationships [49]. Investigations into the different aspects (e.g. lifestyle behaviors, socio-economic, financial) of physical and social health have established the importance of their relationships [50, 51], and as such the following hypotheses are proposed:

### Hypothesis 5a

Physical health has a significant positive effect on social health for managers.

### Hypothesis 5b

Physical health has a significant positive effect on social health for staff.

Physical health encompasses a person’s physical fitness and previous studies have found that achievement motivation is related to physical fitness [52–54] and cardiorespiratory fitness [55, 56]. Although these findings are predominantly based on studies of achievement motivation for sport-related performance, they suggest that achievement motivation is closely linked to physical fitness. Given that the work-related activities in the hospitality industry are highly dependent on physical fitness, it is posited that the physical health of hospitality workers may have an impact on their achievement motivation. Therefore, we propose the following hypotheses:

### Hypothesis 6a

Physical health has a significant positive effect on achievement motivation for managers.

### Hypothesis 6b

Physical health has a significant positive effect on achievement motivation for staff.

Previous studies suggest that building good friendships at a workplace and having a good workplace atmosphere can help improve job performance [57]. Research has also found that the quality of interpersonal relationships (which is important in the hospitality industry) can help moderate the relationship between empathy and job performance [58], which play an essential role in enhancing organizations’ productivity. In addition, it is acknowledged that social support can alleviate job burnout [59], which in turn have an impact on job performance. Thus, the following hypotheses are proposed to explore the effects of social health on job performance.

### Hypothesis 7a

Social health has a significant positive effect on job performance for managers.

### Hypothesis 7b

Social health has a significant positive effect on job performance for staff.

Research studies into social health and achievement motivation have been extensively investigated in different contexts, which have drawn considerable interest and discussion in the academia community. Furthermore, a good peer relationship and friendship can influence achievement motivation [60] and interpersonal relationships [61]. A longitudinal dataset collected by Wu et al. [62] within the first 3 months of college freshmen enrolment in China suggests that achievement motivation can decrease over time and is positively related to a decline in subjective social status. Other cross-sectional studies that explored the relationship between social health and achievement motivation include the impact of stress on achievement motivation and academic performance of senior secondary and university students in India and Iran [63, 64], mediating relationship between gaming disorder, and achievement, social and escapism motivations for German game users [65], the effect of motivations on sports performance in marathon runners and triathlons [66, 67]. In addition, studies have found that interpersonal relationships and social support have a positive effect on the sense of achievement at work [68]. Given the evidence about the relationships between social health and achievement motivation, and the importance of interpersonal interactions in the hospitality industry, the following hypotheses are proposed.

### Hypothesis 8a

Social health has a significant positive effect on achievement motivation for managers.

### Hypothesis 8b

Social health has a significant positive effect on achievement motivation for staff.

There have been numerous cross-sectional studies conducted to examine the relationship between achievement and performance [13, 34, 63]. The key research themes that investigated their relationship have mainly focused in the areas of academic success [69, 70], sports success [71], and performance measurement [72]. There is also evidence to suggest that achievement motivation can influence job performance [14, 16, 73, 74], which leads to our proposed hypotheses as follow:

### Hypothesis 9a

Achievement motivation has a significant positive effect on job performance for managers.

### Hypothesis 9b

Achievement motivation has a significant positive effect on job performance for staff.

**Fig. 1:**
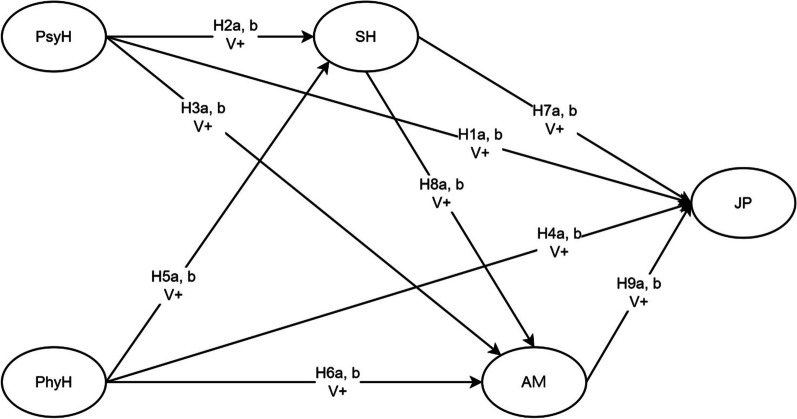
Research framework of this study. Note: Psychological health (PsyH), physical health (PhyH), social health (SH), achievement motivation (AM), and job performance (JP)

With the above proposed hypotheses, a research framework is developed for this study as shown in Fig. 1 below. This study seeks to examine the relationships between psychological health, physical health, social health, achievement motivation, and job performance in the Taiwan hospitality industry from both the managerial and staff perspectives. This study also aims to develop job performance pathway models and contribute to the relevant theory and practical implications.

## Methods

### Participants

The hospitality industry in Taiwan has seen good growth potential over the recent years [75]. In this study, a purposeful sampling technique has been used to select the 10 highest-earning hospitality companies in Taiwan and collected information from their employees. The key eligible criteria to complete the questionnaire were: current employees of the company; must be 18 years and older; must not hold a directorship and above position in the company. The respective company’s public relations department was contacted for assistance to distribute and return of the questionnaires.

### Measures

The final questionnaire consisted of two sections. First section was related to demographic data and included items such as age, gender, position (i.e. manager or staff), educational qualification and their relevance to the hospitality industry. Second section included 23 survey questions related to psychological health (four questions), physical health (three questions), and social health (four questions) were designed using the World Health Organization’s WHOQOL-BREF scale. [76]. The achievement motivation (six questions) was based on the Minnesota Satisfaction Questionnaire (MSQ) [77]. The job performance was developed following Bott et al.’s [78] methodology. All items were measured on a five-point Likert scale (1 = strongly disagree to 5 = strongly agree).

### Procedures

To determine the appropriateness and comprehensibility of the survey questions, six individuals from the hospitality industry management, organizational management, and social behavior had peer reviewed the questionnaire. The questionnaire was subsequently pretested by 50 individuals with a Cronbach’s α value of 0.925. The Cronbach’s α values for psychological health (0.732), physical health (0.783), social health (0.753), achievement motivation (0.782), and job performance (0.865) also exceeded the standard of 0.7 [79].

### Statistical analysis

The data was analyzed using Statistical Package for Social Sciences (SPSS 23) software. Descriptive statistics were used to calculate the total number, percentage, mean, and standard deviation of demographic variables and key variables. Paired-sample *t*-tests were used to determine differences based on the respondents’ gender, hospitality-related or non-hospitality related qualification, and position. The chi-square test was used to determine the differences between the manager and staff in terms of age, education, and hospitality-related or non-hospitality related qualification. Pearson correlation coefficients were used to measure the strength and direction of the relationships between these dimensions. Partial least squares - Structural Equation Modeling (PLS-SEM) is an exploratory or confirmatory method that models structural equations in a small sample study. Past research has pointed out that PLS-SEM could find meaningful information from the minimum sample size of 20 [80]. In view of the number of samples collected, PLS-SEM could help to better understand the job performance pathway model, and the difference between the manager and staff.

## Results

### Descriptive findings

A total of 292 responses were received, of which 61 (20.9%) were from managers and 231 (79.1%) were from staff. The Cronbach α value for the overall scale of the returned questionnaires was 0.907, which was evident for internal reliability of the questionnaires [79]. The Cronbach α values for social health (0.765), achievement motivation (0.835), and job performance (0.855) variables had also exceeded the standard value of 0.7. However, the Cronbach α values for psychological health (0.688), and physical health (0.687) variables were below 0.7, though still above the acceptable standard of 0.5 [81] for internal reliability.

The descriptive statistics and independent sample *t*-test of gender, education level and work position in the study are shown in Table 1. Findings showed that there were slightly more males than females, and one respondent did not specify the gender. Of these respondents, 52.4% had attained a non-hospitality related education qualification, and the remaining 46.6% were hospitality related. There were three respondents who did not indicate the type of education qualification. Independent sample *t*-tests were conducted on the five key dimensions (i.e. psychological health, physical health, social health, achievement motivation, and job performance) with gender, educational qualification (i.e. hospitality related or non-hospitality related), and position (i.e. manager or staff). Although results revealed no significant differences in all aspects (except for psychological health by gender), the pathways were different depending on the results of the pathway analysis.

**Table 1:** Results of the *t*-tests by gender, educational qualification, and position

Variables	Frequency		PsyH				PhyH				SH				AM				JP			
			M	SD	*t*	*p*	M	SD	*t*	*p*	M	SD	*t*	*p*	M	SD	*t*	*p*	M	SD	*t*	*p*
*Gender*																						
Female	142	3.24		0.70	2.24	0.03	3.53	0.69	0.47	0.64	3.60	0.62	0.84	0.40	3.50	0.64	0.55	0.59	3.81	0.54	0.24	0.81
Male	149	3.01		0.56			3.49	0.61			3.55	0.57			3.46	0.53			3.80	0.53		
*Educational Qualification*																						
Hospitality related	136	3.11		0.63	-1.04	0.30	3.50	0.64	-0.04	0.97	3.62	0.63	1.36	0.17	3.52	0.62	1.55	0.12	3.84	0.56	1.10	0.27
Non-hospitality related	153	3.19		0.64			3.51	0.66			3.52	0.57			3.42	0.56			3.77	0.51		
*Position*																						
Manager	61	3.27		0.60	1.66	0.10	3.56	0.50	0.76	0.45	3.63	0.68	0.84	0.40	3.56	0.65	1.18	0.24	3.83	0.56	0.43	0.67
Staff	231	3.12		0.64			3.50	0.69			3.55	0.58			3.45	0.57			3.80	0.52		

Psychological health (PsyH), physical health (PhyH), social health (SH), achievement motivation (AM), and job performance (JP)

Most of the respondents were aged between 24-28 years old. There was one respondent who did not specify the age. To understand the compositional differences between managers and staff, an analysis of their age, educational qualification and their relevance to a hospitality related qualification was conducted using a chi-square test. Results (please refer to Table 2) showed that there was a significant difference in terms of age between managers and staff where there was a higher proportion of managers aged between 29-33, 34-38, and 38 years or older. In contrast, staff who were younger than 23 years old had a higher proportion instead.

**Table 2:** Age distribution of respondents

Age (years)	Age groups					Total	*χ*^*2*^	*p*
	< 23	24–28	29–33	34–38	38 +			
Manager	6 (9.8%)	27 (44.3%)	15 (24.6%)	5 (8.2%)	8 (13.1%)	61	27.684	< 0.001***
Staff	76 (33.0%)	103 (44.8%)	37 (16.1%)	10 (4.3%)	4 (1.7%)	230		
Total	82 (28.2%)	130 (44.7%)	52 (17.9%)	15 (5.2%)	12 (4.1%)	291		

One person who did not disclose their age was excluded from this analysis

Majority of the respondents had a university degree qualification and there was a significant difference between managers and staff. As shown in Table 3, there was a higher proportion of managers who had a high school, and master’s degree and higher qualification, whereas the proportion of staff who had a university, and junior high school and lower qualification was higher.

**Table 3:** Educational qualification of respondents

Attributes	Educational qualifications				Total	*χ*^*2*^	*p*
	Junior high school and lower	High school	University or junior college	Master’s degree and higher			
Manager	8 (13.1%)	11 (18.0%)	39 (63.9%)	3 (4.9%)	61	11.970	0.007**
Staff	49 (21.2%)	22 (9.5%)	159 (68.8%)	1 (0.4%)	231		
Total	57 (19.5%)	33 (11.3%)	198 (67.8%)	4 (1.4%)	292		

In relation to the types of the educational qualification (i.e. hospitality related versus non-hospitality related), results revealed no significant difference between managers and staff. As outlined in Table 4, there was a higher proportion of manager holding a non-hospitality related qualification, whereas staff had a higher proportion in holding a hospitality related qualification.

**Table 4:** Relevance of educational qualification of respondents

Attributes	Have a hospitality related qualification?		Total	*χ*^*2*^	*p*
	Yes	No			
Manager	25 (41.0%)	36 (59.0%)	61	1.145	0.285
Staff	111 (48.7%)	117 (51.3%)	228		
Total	136 (47.1%)	153 (52.9%)	289		

Three respondents did not answer the relevance of their educational qualification and were excluded

The results of each question under the key variables and their mean and standard deviation are shown in Table 5. The table also presents the scores of the manager and staff for each item under each key variable and the average value of the overall variable. In addition, results of the ANOVA analysis conducted showed that age and education had no significant difference in all the key variables.

**Table 5:** Key variables survey results

Question	Manager (n = 61)			Staff (n = 231)	
	Mean		SD	Mean	SD
*Psychological health*					
PsyH1. Do you enjoy life?	3.02		0.70	2.89	0.92
PsyH2. Can you concentrate well?	3.60		0.97	3.39	0.96
PsyH3. Are you satisfied with your appearance?	3.02		0.85	2.77	0.79
PsyH4. Are you satisfied with yourself?	3.46		0.87	3.44	0.87
Overall psychological health	3.27		0.60	3.12	0.64
*Physical health*					
PhyH1. Are you satisfied with your ability to perform daily activities?	3.54		0.65	3.39	0.81
PhyH2. Are you satisfied with your ability to do your job?	3.43		0.78	3.46	0.79
PhyH3. Are you good at moving around?	3.70		0.82	3.64	0.95
Overall physical health	3.56		0.50	3.50	0.69
*Social health*					
SH1. Recognition by supervisors, peers, and subordinates	3.41		0.94	3.37	0.76
SH2. Opportunities to build good friendships	3.61		0.86	3.54	0.74
SH3. Being valued and respected by colleagues	3.56		0.74	3.55	0.70
SH4. Authority granted at work	3.93		0.93	3.76	0.84
Overall social health	3.63		0.68	3.56	0.58
*Achievement motivation*					
AM1. Being able to realize one’s life ambitions		3.80	0.79	3.60	0.70
AM2. Opportunities to use knowledge and skills at work		3.80	0.81	3.57	0.72
AM3. Being able to provide opportunities for professional autonomy		2.97	1.08	3.13	0.91
AM4. Future and prospects of one’s position		3.56	0.79	3.43	0.87
AM5. Social status of one’s position		3.67	0.72	3.59	0.69
AM6. Satisfaction and self-sufficiency from work		3.51	0.87	3.39	0.74
Overall achievement motivation		3.55	0.65	3.45	0.57
*Job performance*					
JP1. I have the operational ability to do the job	3.98		0.65	3.86	0.66
JP2. Demonstrating expertise in the workplace	3.80		0.87	3.71	0.62
JP3. Meeting the requirements of the job	4.05		0.82	4.04	0.64
JP4. Maintaining good behavior and performance at work	3.83		0.69	3.86	0.72
JP5. Having good daily habits for work	3.57		0.76	3.69	0.72
JP6. Mastering the process of production or service at work	3.74		0.68	3.63	0.70
Overall job performance	3.86		0.56	3.80	0.52

### Correlation analysis

As shown in Table 6, results of the correlation analysis for the manager showed a significant positive correlation between all five dimensions tested. All these dimensions were correlated with varying degree of significance, with the strongest relationship shown between social health and achievement motivation (*p*=0.814***), and this was followed by physical health and psychological health (*p*=0.683***), job performance and physical health (*p*=0.611***), job performance and psychological health (*p*=0.606***), social health and physical health (*p*=0.459***), achievement motivation and physical health (*p*=0.422***), job performance and social health (*p*=0.396**), achievement motivation and psychological health (*p*=0.387**), job performance and achievement motivation (*p*=0.378**), and social health and psychological health (*p*=0.319*).

**Table 6:** Correlation matrix between the five key dimensions for the manager

	PsyH	PhyH	SH	AM	JP
PsyH	1.000				
PhyH	0.683***	1.000			
SH	0.319*	0.459***	1.000		
AM	0.387**	0.422**	0.814***	1.000	
JP	0.606***	0.611***	0.396**	0.378**	1.000

Psychological health (PsyH), physical health (PhyH), social health (SH), achievement motivation (AM), and job performance (JP)

***p < 0.001 two-tailed, **p < 0.01 two-tailed, *p < 0.05 two-tailed

Results (please see Table 7) of the correlation analysis for the staff suggested that there was a significant positive correlation between all five dimensions and the significance *p*-values were less than 0.001. The strongest relationship was between social health and achievement motivation (*p*=0.681***), and this was followed by physical health and psychological health (*p*=0.588***), achievement motivation and psychological health (*p*=0.515***), job performance and psychological health (*p*=0.458***), job performance and achievement motivation (*p*=0.453***), job performance and physical health (*p*=0.429***), job performance and social health (*p*=0.427***), social health and psychological health (*p*=0.398***), social health and physical health (*p*=0.380***), and achievement motivation and physical health (*p*=0.350***).

**Table 7:** Correlation matrix between the five key dimensions for the staff

	PsyH	PhyH	SH	AM	JP
PsyH	1.000				
PhyH	0.588***	1.000			
SH	0.398***	0.380***	1.000		
AM	0.515***	0.350***	0.681***	1.000	
JP	0.458***	0.429***	0.427***	0.453***	1.000

Psychological health (PsyH), physical health (PhyH), social health (SH), achievement motivation (AM), and job performance (JP)

***p < 0.001 two-tailed

### Path analysis and PLS-SEM

The impact factors for both the manager’s and staff’s job performance were analyzed using SmartPLS2.0. According to the PLS results, different job performance paths were identified for the manager and staff respectively.

As shown in Table 8, the PLS results for the manager revealed that the AVE values of the social health, achievement motivation, and job performance dimensions were all greater than 0.5, indicating that they reached a level of convergent validity [82]. While the AVE values of the physical health and psychological health dimensions were less than 0.5, but they were still greater than 0.4, which is considered acceptable [82]. The Cronbach’s α value of the five dimensions were all above the 0.5 confidence level. The R^2^ values for social health, achievement motivation, and job performance were 0.2377, 0.5372 and 0.4237 respectively.

**Table 8:** Indicator data of the PLS analysis for the manager

	AVE	CR	R^2^	Cronbach’s α
PhyH	0.4981	0.7417		0.5154
PsyH	0.4883	0.7854		0.6524
SH	0.7181	0.9105	0.2377	0.8688
AM	0.6731	0.9249	0.5372	0.9025
JP	0.6044	0.9010	0.4237	0.8675

Psychological health (PsyH), physical health (PhyH), social health (SH), achievement motivation (AM), and job performance (JP)

**Fig. 2:**
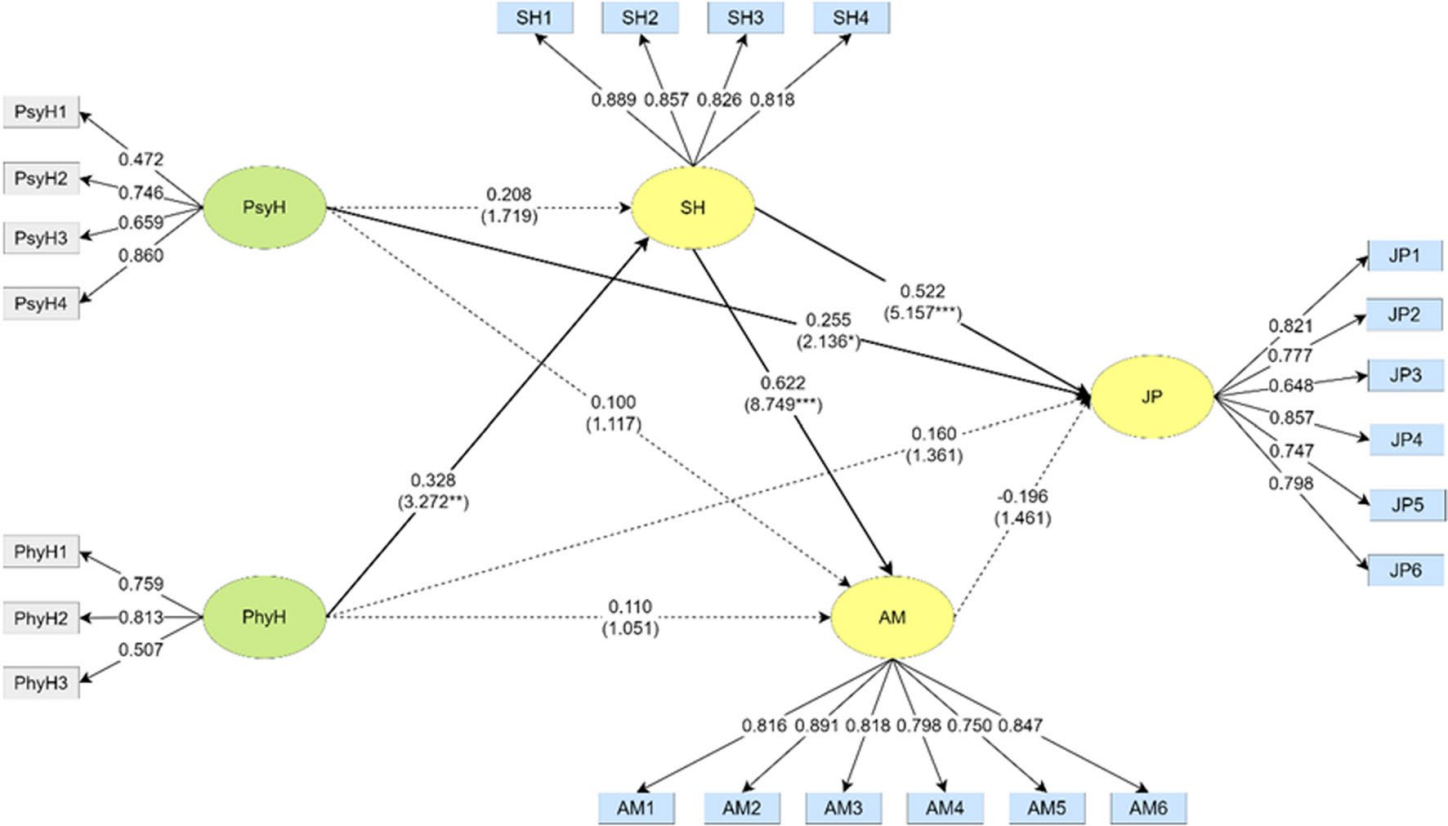
Path coefficients of PsyH, PhyH, SH, AM, and JP in the model for the manager. *p < 0.05, **p < 0.01, ***p < 0.001. Note: Psychological health (PsyH), physical health (PhyH), social health (SH), achievement motivation (AM), and job performance (JP)

A structural equation model (as shown in Fig. 2) was developed to identify the relationship between the paths of each dimension for the manager, and the *t*-value of the pathways was obtained by Bootstrapping to examine its significance level. The psychological health of the manager had a significant positive predictive effect on job performance. In addition, the social health of the manager had a significant positive predictive effect on job performance and a significant positive direct effect on achievement motivation. The physical health of the manager had a significant positive direct effect on social health, and social health was also a mediating variable between physical health and job performance. The direct and indirect effects of significant paths among managers are shown in Table 9.

**Table 9:** Direct and indirect effects of the manager group

Path	Direct effect	Indirect effect
PsyH → JP	0.255(2.136*)	
PhyH → SH → AM		0.204(2.943**)
PhyH → SH → JP		0.171(2.579**)

The PLS results (please see Table 10) for the staff showed that the AVE values for the five dimensions were all greater than 0.5, which suggested a level of convergent validity. The Cronbach’s α value of the five dimensions were all above the 0.5 confidence level. The R^2^ values for social health, achievement motivation, and job performance were 0.2606, 0.5965 and 0.3439 respectively.

**Table 10:** Indicator data of the PLS analysis for the staff

	AVE	CR	R^2^	Cronbach’s α
PhyH	0.6186	0.8280		0.6902
PsyH	0.5151	0.8081		0.6935
SH	0.6798	0.8943	0.2606	0.8416
AM	0.5949	0.8979	0.5965	0.8633
JP	0.9321	0.9114	0.3439	0.8828

Psychological health (PsyH), physical health (PhyH), social health (SH), achievement motivation (AM), and job performance (JP)

**Fig. 3:**
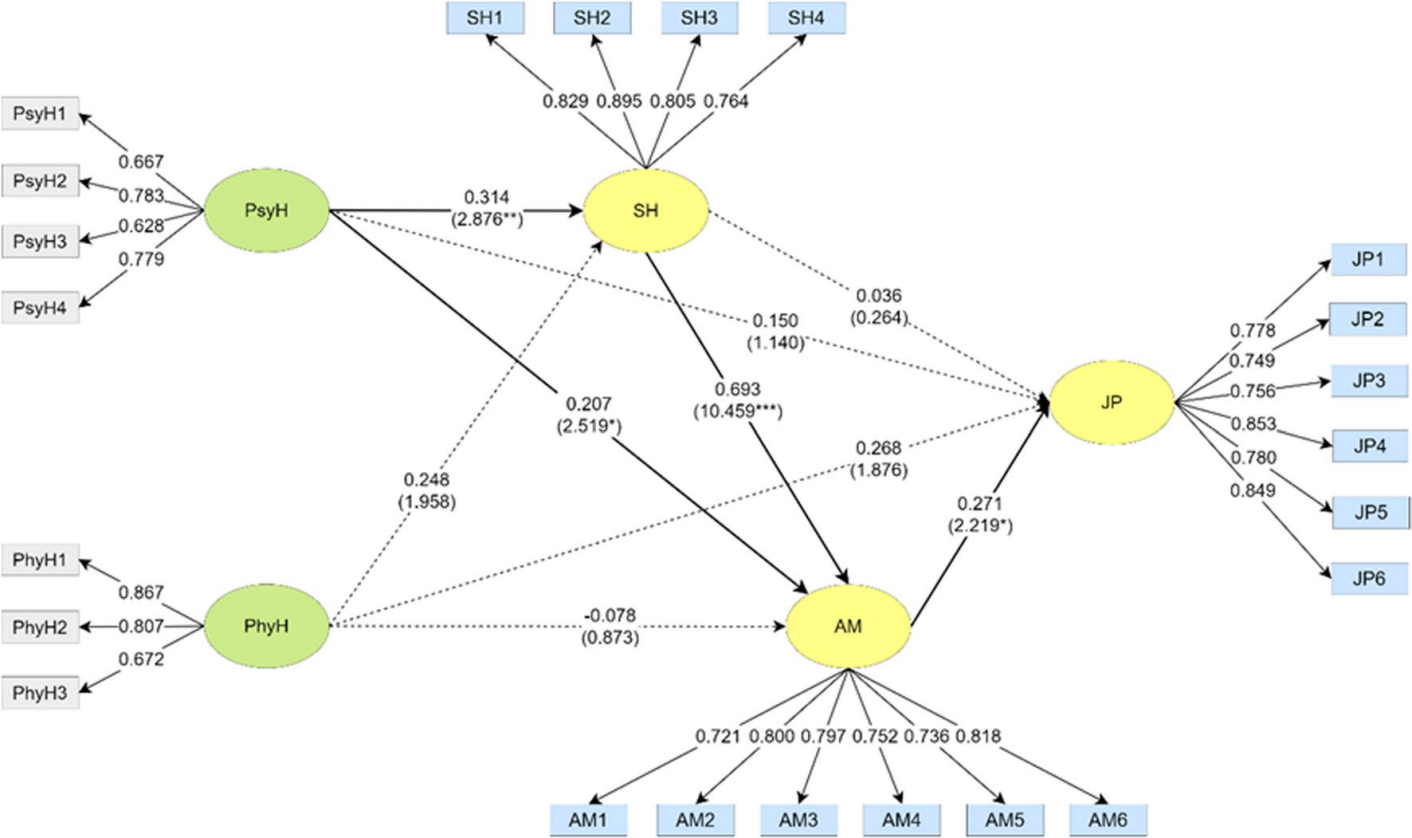
Path coefficients of PsyH, PhyH, SH, AM, and JP in the model for the staff group. *p < 0.05, **p < 0.01, ***p < 0.001. Note: Psychological health (PsyH), physical health (PhyH), social health (SH), achievement motivation (AM), and job performance (JP)

A structural equation model (as shown in Fig. 3) for the staff was developed to identify the relationship between the paths of each dimension, and the *t*-value of the pathways was obtained by Bootstrapping to examine its significance level. The psychological health of staff had a significant positive effect on social health and achievement motivation. The social health of staff also had a significant positive effect on achievement motivation. Achievement motivation was the only dimension that directly influenced job performance. The direct and indirect effects of significant paths among staff are shown in Table 11.

**Table 11:** Direct and indirect effects of the staff group

Path	Direct effect	Indirect effect	Total effect	
			AM	JP
PsyH → AM	0.207(2.545*)		0.425(3.760***)	
PsyH → SH → AM		0.218(2.744**)		
PsyH → AM → JP		0.056(1.601)		0.114(1.804)
PsyH → SH → AM → JP		0.058(1.568)		

## Discussion

This study developed a structural model to explore the pathways of HRQoL (i.e. psychological health, physical health, and social health), achievement motivation and job performance between manager and staff. This can potentially help hospitality organizations to operate more smoothly and achieve better organizational performance and productivity. Results have supported eight of the 18 proposed hypotheses, and of which four (i.e. H1a, H5a, H7a, and H8a) relate to the manager’s pathway model, while the other four (i.e. H2b, H3b, H8b, and H9b) are in the staff’s pathway model. The chi-squared test showed that there was a higher proportion of the manager who were older and with a higher education qualification than the staff. However, the ANOVA tests conducted did not reveal any significant differences in the five key dimensions (i.e. physical health, psychological health, social health, achievement motivation, and job performance) by age and educational qualification.

### Influence of psychological health

Findings suggested that psychological health was a key factor that could directly affect job performance in the manager’s pathway structure, and this echo previous studies that psychological health could affect job performance [25, 26]. However, psychological health did not have a direct effect on social health and achievement motivation. In addition, psychological health did not indirectly affect job performance through social health and achievement motivation.

On the other hand, the pathway structure for staff indicated that psychological health did not directly affect their job performance, but instead could directly affect their social health and achievement motivation. This was consistent with previous studies, which confirmed psychological health contributed to social interactions [29] and was closely related to achievement motivation [35].

### Influence of physical health

Findings indicated that there was no significant positive relationship between physical health and job performance and achievement motivation in the manager’s job performance pathway. However, there was a significant positive effect of physical health on social health, and this aligned with prior studies [10, 46].

In terms of the staff’s job performance pathway structure, physical health did not have any significant positive effects on job performance, social health, and achievement motivation. Although previous studies have argued that physical health could be related to job performance, findings in this study suggested otherwise.

### Influence of social health

Findings indicated that social health had a direct positive effect on achievement motivation and job performance in the manager’s job performance pathway structure, which supported the argument that interpersonal relationships affect achievement motivation [60, 61] and those interpersonal relationships could contribute towards job performance [57]. Social health was also influenced by physical health, and it was not significantly related to psychological health. This suggests that when the manager displays better social health, it can lead to enhancing job performance and achievement motivation.

For staff’s performance pathway structure, social health also presented a significant positive effect on achievement motivation. This supported the contention that interpersonal relationships affect achievement motivation [60, 61]. However, staff’s social health did not exhibit a significant positive effect on job performance. Unlike the manager’s, staff’s social health was not directly influenced by physical health but instead was positively influenced by psychological health.

### Influence of achievement motivation

Although findings showed that achievement motivation was positively influenced by social health at a significant level in the manager’s job performance path structure, it did not have a significant positive effect on job performance. This suggests that while better social health could contribute towards enhanced job performance, better achievement motivation does not lead to a similar outcome.

With regards to the staff’s job performance pathway structure, achievement motivation had a significant direct positive effect on job performance, as well as influence from psychological health and social health. Results for staff’s job performance path structure supported numerous studies that suggested achievement motivation could influence job performance [14, 16, 73].

By combining the HRQoL and achievement motivation theoretical framework, we investigated how psychological health, physical health, social health, and achievement motivation affect the job performance of the manager and staff in Taiwan’s hospitality industry. We filled a research gap by constructing a pathway model to further identify the specific pathways and degree of influence on the employees’ (including the manager and staff) job performance in the hospitality industry.

### Influence on manager and staff

In terms of organizational management, ensuring a manager’s psychological health could lead to better job performance. A good physical health could also contribute towards better interpersonal relationships and satisfaction, and social health that help enhance job performance. Psychological health of staff was equally important because it contributed to their interpersonal relationships, and achievement motivation. Furthermore, achievement motivation of staff could enhance their job performance. Although the findings suggested that physical health did not affect other factors, job performance should not be taken to imply that the physical health of staff should be ignored in the workplace because physical health might be the most neglected factor in the workplace for staff, putting them at a higher risk of overwork and workplace hazards [83–85].

It should be noted that staff’s job performance was mainly influenced by achievement motivation and improving this aspect should help improve job performance. In addition, maintaining the psychological health of staff, keeping good interpersonal interactions throughout the organization, creating a good working atmosphere, and enhancing achievement motivation could also help improve staff job performance. In subordinate-to-superior support, staff-level employees could help the manager to create good interpersonal relationships and social health in the workplace to improve overall job performance and build good cooperative interactions.

## Conclusions

In conclusion, this study has investigated the relationships between psychological health, physical health, social health, achievement motivation, and job performance among the manager and staff in the hospitality industry in Taiwan. In addition, pathway models have been developed to better understand the influences on the manager’s and staff’s job performance.

### Implications, limitations and future research

By combining the HRQoL and achievement motivation theoretical framework, this study provided new insights on how psychological health, physical health, social health, and achievement motivation affected job performance of the manager and staff in Taiwan’s hospitality industry. These results could potentially influence government and corporate management policies, such as the need to improve the psychological, physical, and social health of hospitality employees in terms of laws, policies, and corporate strategies. The results could also encourage companies to consider the differences between the manager and staff with respect to communication and information processing, to enhance employees’ overall job performance through improved organizational management and the establishment of good communication channels.

Although this study has investigated the job performance and influencing factors of the manager and staff from 10 of the highest-earning hospitality companies in Taiwan, our results are not fully representative of the entire national or global hospitality workforce. This is a single source of information study and future research should consider targeting a more comprehensive sample and size to provide a better representation of the population at large. Further comparisons between other countries are also needed to determine any similarities or differences in this regard. Finally, this research is a cross-sectional quantitative study, and a longitudinal study is needed to determine possible long-term impacts and developments.

## Data Availability

Data supporting the findings of this study is available upon reasonable request from the corresponding author.
